# Impact of El Niño/Southern Oscillation on Visceral Leishmaniasis, Brazil

**DOI:** 10.3201/eid0809.010523

**Published:** 2002-09

**Authors:** Carlos Roberto Franke, Mario Ziller, Christoph Staubach, Mojib Latif

**Affiliations:** * Federal University of Bahia, Bahia, Brazil; † Federal Research Centre for Virus Diseases of Animals, Wusterhausen, Germany; ‡ Max-Planck-Institut für Meteorologie, Hamburg, Germany

**Keywords:** visceral leishmaniasis, El Niño, Bahia, Brazil

## Abstract

We used time-series analysis and linear regression to investigate the relationship between the annual Niño-3 index from 1980 to 1998 and the annual incidence of visceral leishmaniasis (VL) in the State of Bahia, Brazil, during 1985–1999. An increase in VL incidence was observed in the post-El Niño years 1989 (+38.7%) and 1995 (+33.5%). The regression model demonstrates that the previous year’s mean Niño-3 index and the temporal trend account for approximately 50% of the variance in the annual incidence of VL in Bahia. The model shows a robust agreement with the real data, as only the influence of El Niño on the cycle of VL was analyzed. The results suggest that this relationship could be used to predict high-risk years for VL and thus help reduce health impact in susceptible regions in Brazil.

Visceral leishmaniasis (VL) is a widespread parasitic disease in the Old and New Worlds, with a global incidence of 500,000 new human cases each year. VL is the most severe clinical form within the leishmaniasis complex, which is endemic in 88 countries with an at-risk population of approximately 350 million (1). In Brazil, VL affects both humans and animals and is caused by *Leishmania chagasi*, a flagellate protozoan transmitted by the sand fly *Lutzomyia longipalpis* (2). The disease occurs mainly in malnourished young children and is frequently fatal if untreated (3,4). Periodic epidemic waves of VL, observed mainly in northeastern Brazil, have been associated with human migrations to urban areas after long periods of drought (5–8). In this region, El Niño events are related to unusually dry conditions, widespread food scarcity, and migration (9–11). El Niño periods in 1982–1983, 1986–1987, 1991–1993, and 1997–1998 coincided with long droughts recorded by the Superintendence for the Development of the Northeast Brazil (SUDENE). Data from the State of Bahia, analyzed in our study, show that 247 municipalities were affected during the strong El Niño of 1997–98, mainly in the semi-arid inland region, where approximately 200,000 people were included in SUDENE’s emergency program, at a cost of an estimated US $62 million.

 El Niño is the strongest interannual climate fluctuation worldwide, characterized by a large-scale warming of the eastern and central equatorial Pacific Ocean. El Niño (also known as El Niño/Southern Oscillation) can be understood as the warm phase of an irregular cycle with an average frequency of 3–4 years. Each event typically lasts for approximately a year, with the peak warming in boreal winter (December–February) and the following spring (March–May) (12). Some studies provide strong evidence of the relationship between El Niño and increased epidemic risk of vector-borne diseases in distinct regions throughout the world (13,14). This observation is especially true for malaria (15–18). We report the early results of our analysis of the relationship between the El Niño cycle and VL in Brazil.

## Methods

The State of Bahia (pop. 13,093,243, in the 2000 census) is situated on the northeast Atlantic coast of Brazil. Its area is 567,295 km^2^, divided into 415 municipalities. The annual number of VL cases from 1985 to 1999 was obtained from the Public Health Secretary of the State of Bahia. In this period in Bahia, 12,413 cases of VL were reported by using a passive case-detection procedure. Diagnosis was based on clinical and epidemiologic features, confirmed by immunofluorescence assay or detection of parasites by examination of smears of bone marrow, lymph node, or splenic aspirate.

For our analysis, we used the mean monthly Niño-3 index for the years 1980 to 1998. This index is the tropical Pacific sea surface temperature (SST) anomaly averaged over 150°W–90°W, 5°N–5°S, obtained from the Hadley Centre dataset (19). To evaluate the relationship between El Niño and VL and to quantify the delay of its possible impact, we calculated the cross-correlation function between the annual incidences of VL per 10,000 inhabitants from 1985 to 1999 and the 12-month moving average of the mean monthly Niño-3 index. The linear temporal trend was removed from both variables before analysis. Additionally, to model the observed dependence, we calculated a linear regression between the annual incidence of VL, the year (to consider the linear temporal trend), and the mean annual Niño-3 index 12 months previously.

## Results

**Figure 1:**
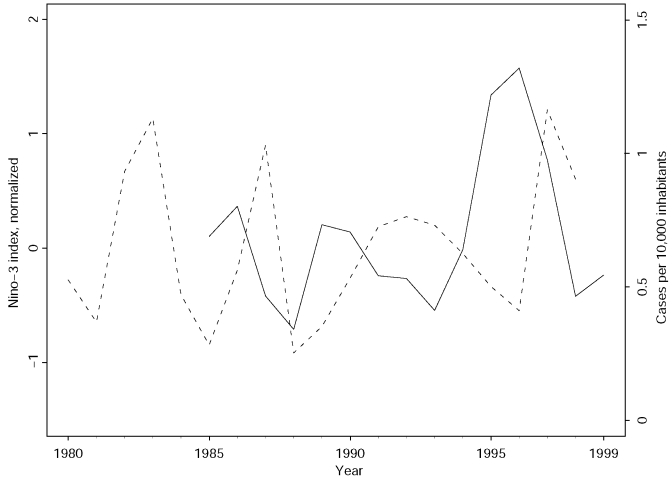
The Niño-3 index and the incidence of VL in the State of Bahia, Brazil, on a yearly basis. The broken line is the normalized mean annual Nino-3 index, 1980–1998. The solid line shows the annual number of cases of VL per 10,000 inhabitants during 1985–1999.

 We observed that, despite the wide territorial area of Bahia and the complex epidemiologic nature of VL, the cycle of the annual incidence appears to be narrowly associated with both the frequency and duration of El Niño episodes. Low incidence levels coincide with the occurrence of El Niño, and an increase in incidence occurs after such climatic events (Figure 1). We found extreme increases of the incidence in relation to the 5-year moving average for the years 1989 (+38.7%) and 1995 (+33.5%).

**Figure 2:**
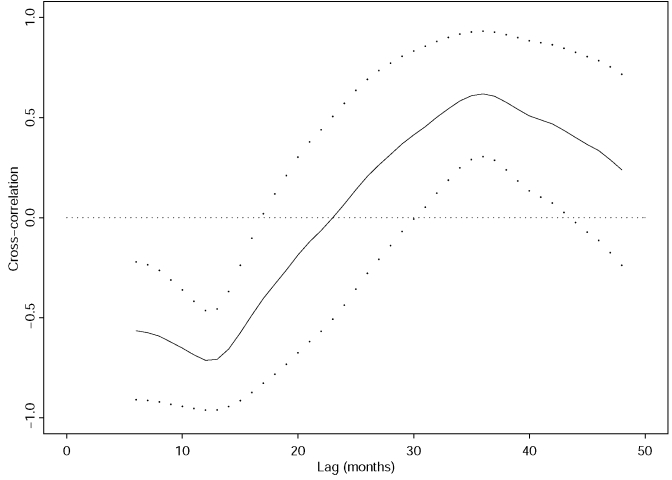
Cross-correlation function between the annual incidences of visceral leishmaniasis from 1985 to 1999 and the 12-month moving average of the mean monthly Niño-3 index (solid line). Broken lines are the corresponding 95% pointwise confidence intervals.

The cross-correlation function between the annual incidences of VL and the 12-month moving average of the mean monthly Niño-3 index has its strongest negative correlation at a lag of 12 months and its strongest positive correlation at a lag of 36 months (Figure 2). The positive correlation may, in part, result from a broad minimum of the autocorrelation function of the mean monthly Niño-3 index at lags from 16 to 28 months, with autocorrelation values of -0.54 < correlation coefficient (r) < -0.45.

**Figure 3:**
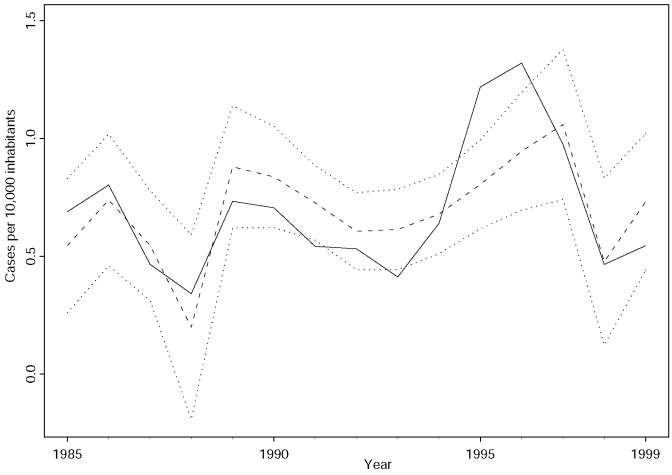
Result of the regression model. The figure represents the annual number of cases of visceral leishmaniasis per 10,000 inhabitants during 1985 to 1999 (solid line), the fitted regression model (broken line), and the corresponding 95% confidence limits (dotted lines).

The results of the regression model (root square error [RSE] = 0.209, adjusted coefficient of determination [adj. R^2^] = 0.465, F test with 2 and 12 degrees of freedom [F_2;12_] = 7.081, p = 0.009 demonstrate that the mean Niño-3 index 12 months previously and the temporal trend can account for approximately 50% of the variance in the annual incidence of VL in Bahia (Figure 3). The model shows a robust agreement with the real data, considering that only the influence of El Niño on the variance of VL incidence was analyzed.

## Discussion

Our results show that El Niño episodes are related to variation in the annual incidence of VL in the State of Bahia and suggest that an El Niño–based early warning system for VL may help reduce the health impact of the disease in susceptible regions in Brazil. According to our results, the annual incidence of VL in Bahia tends to reach its lowest level in the first year after El Niño episodes and begins to increase in the second year after El Niño. The delay of this correlation pattern is unusual in light of the well-studied association between malaria and El Niño reported in different regions of the world. Increases in malaria incidence have been reported to accompany El Niño episodes or occur in the year immediately following such episodes (15–18).

Because of the multifactorial interactions involved in this complex system, a simple deterministic explanation for the interannual correlation between El Niño and VL is not possible. However, some climatic and epidemiologic behaviors related to VL could be helpful in drafting a preliminary assumption about pathways by which El Niño can affect the temporal distribution pattern of the disease. In the New World, >90% of VL cases have been reported in Brazil, mainly in the semi-arid northeast region (2,20), where dry and rainy seasons are clearly defined. Furthermore, in this region, evidence indicates that the population density of the sand fly vector is low during the dry season and increases after the end of the rainy season (December–April), reaching its highest density level around May (5,21). The resulting increase in transmission intensity during this seasonal peak of vector density leads to an increase in the reported incidence after this period (4,5,21). In the semi-arid region of Bahia, El Niño episodes coincide with the rainy season and negate its effect, causing a prolonged drought by connecting the previous dry season, the period of El Niño, and the subsequent dry season. We hypothesize that, in view of the negative influence of dry conditions on the vector density, the long droughts triggered by El Niño events could be expected to be accompanied by prolonged low vector density and low transmission intensity. These exceptional circumstances most likely contribute to a gradual increase in the potential risk of some related epidemiologic factors, such as waning herd immunity, increase in the susceptible population in the endemic areas (as a result of new births and nonimmune immigrants), and displacement of human and animal carriers to regions with populations that lack a protective immunity (4,20). Drought-related food scarcity and growing malnutrition also increase the susceptibility to VL within these risk populations (4,22).

The rainy season at the beginning of the first year after El Niño is followed by an increase in vector density (around May), which triggers a severe increase in infection rate in high-risk populations. However, the incubation period (2–6 months) (23), added to the time from the onset of VL symptoms until diagnosis of the disease (approximately 3 months, according to data from the Federal Health Foundation of Bahia [data not shown]), shifts the reporting of most new cases into the second year after El Niño. Although this preliminary assumption provides a plausible explanation of the lag time and intensity of the teleconnection pattern between El Niño and VL cycle in Bahia, the occurrence of distinct climate and vector seasonality patterns on the regional level could lead to differences in the lag time previously discussed.

In accordance with our assumption, the divergent regression fits of the model observed for the years 1991, 1993, 1995, and 1996 most likely reflect the influence of the exceptionally long El Niño period from 1991 to 1993, which led to a lasting reduction in transmission intensity. However, in accordance with our assumption, the annual incidence of VL increased from 1994 to 1996. The divergent regression fits observed for the years 1995 and 1996 might have been caused by the sum of two factors: the critical increase in the related risk factors following a longer El Niño episode and improvement of the surveillance system. The latter occurred in 1994, when new endemic areas were included. This expanded surveillance probably facilitated better reporting of this epidemic phase of the VL cycle in Bahia. However, the low incidence reported for 1997 and 1998 (El Niño period) shows a reduced impact of this new system on the detection of new VL cases. Statistically based forecast models also failed to predict the evolution of this climatic anomaly during 1990–1993 in northeast Brazil (11). Within our limited time-series of VL data, only one longer El Niño period is included. Indeed, our model underestimates the intensity of the impact of this atypical El Niño event on the occurrence of VL. The real impact of this modified surveillance system can only be evaluated through a comparative analysis with future data.

The extreme changes in the annual incidence pattern of VL associated with the atypical occurrence of El Niño during 1991–1993 do, however, provide a possible example of the potential impact of future variations of the El Niño cycle on public health. This observation is especially interesting given the expected rising frequency of El Niño following the continuous increase of greenhouse-gas concentrations in the atmosphere (24).

The results presented here provide the first evidence of the relationship between the El Niño cycle and VL. Greater global understanding of this complex relationship, particularly of the impact of El Niño on the population dynamics of humans, animal hosts, and sand fly vectors, could provide additional tools to predict epidemic risk. The ability to forecast VL on the basis of El Niño activity about 12 months before outbreaks could permit preventive improvements on public health infrastructure, including access to financial resources, technical knowledge, active disease surveillance, and targeted vector control to reduce the risk of the increased transmission. Such forecasts would reduce disease and death from visceral leishmaniasis in susceptible regions.
